# An Association Between Fetal Subarachnoid Space and Various Pathologies Using MR Imaging

**DOI:** 10.3390/diagnostics14222535

**Published:** 2024-11-13

**Authors:** Lior Onn-Margalit, Tal Weissbach, Michal Gafner, Shalev Fried, Ayelet Wandel, Tomer Ziv-Baran, Eldad Katorza

**Affiliations:** 1 Arrow Program for Medical Research Education, Sheba Medical Center, Ramat-Gan 5262000, Israel; tal.weissbach@sheba.health.gov.il (T.W.); eldad.katorza@sheba.health.gov.il (E.K.); 2 Department of Obstetrics and Gynecology, Sheba Medical Center, Ramat-Gan 5262000, Israel; 3 Faculty of Medicine, Tel Aviv University, Tel Aviv 6997801, Israel; michalgurevitch@gmail.com (M.G.); shalev.fried@sheba.health.gov.il (S.F.); ayeletw@shamir.gov.il (A.W.); zivtome@tauex.tau.ac.il (T.Z.-B.); 4 Department of Pediatrics B, Schneider Children’s Medical Center of Israel, Petach Tikva 4917002, Israel; 5 The Goldschleger Eye Institute, Sheba Medical Center, Ramat-Gan 5262000, Israel; 6 Department of Diagnostic Radiology, Shamir Medical Center, Be’er Yaakov 7033001, Israel; 7 The Gertner Institute for Epidemiology & Health Policy Research, Sheba Medical Center, Ramat-Gan 5262000, Israel

**Keywords:** subarachnoid space, macrocephaly, microcephaly, magnetic resonance imaging, fetal growth retardation

## Abstract

**Background/Objectives**: This study aimed to explore a relationship between the fetal subarachnoid space (SAS) width and various fetal pathologies, employing fetal brain MRI scans. **Methods**: A retrospective collection of fetal brain MRI scans of 78 fetuses was performed with sonographic indications of microcephaly, macrocephaly, or fetal growth restriction (FGR), during a 7-year period at a single tertiary center. The SAS width (named the SAS index) was manually measured in millimeters in ten specific anatomical locations (four in the axial plane and six in the coronal plane), and then converted to centiles by comparing it to (previously collected) data of apparently healthy fetuses. We evaluated the median SAS centiles using the Kruskal–Wallis and Mann–Whitney U tests for statistical comparison. **Results**: Seventy-eight subjects (mean gestational age of MRI scan 34.2 ± 2.2 weeks) were evaluated. The median SAS centiles were consistently higher in the macrocephaly group compared to the microcephaly group in all ten anatomical locations (statistically significant except coronal left inferior temporal gyri). Most pronounced difference was displayed in the insula gyri (axial and coronal). The median SAS centiles were higher in the microcephaly group when compared with FGR across all ten anatomical locations (all were statistically significant except for coronal frontal and insula gyri), and the maximal difference was found in the frontal gyri of both planes. The median SAS indexes (IQR) of the three groups in millimeters: macrocephaly 91.55 (86.35–101.05), microcephaly 59.46 (50.00–66.91), and FGR 53.21 (49.71–59.10), *p* < 0.001. **Conclusions**: We found a statistically significant association between the fetal subarachnoid space and various fetal pathologies: macrocephaly, microcephaly, and FGR.

## 1. Introduction

The development of the human brain begins in early fetal life, evolving continuously with each week of gestation. The brain and spinal cord are enveloped by three membranes (known as the meninges) that serve to protect and support these organs. The subarachnoid space (SAS) is a key anatomical structure in this nervous system, a rather potential space that lies between the arachnoid membrane and pia mater, two of the three meningeal layers [1]. This space is filled with cerebrospinal fluid that plays several vital roles, including waste removal and shock absorption [2,3]. Additionally, the SAS contains blood vessels that supply the brain and spinal cord, thus maintaining a stable environment for these organs and enabling brain health and function. Importantly, the SAS is not only crucial for protecting and nourishing the brain, but also serves as a marker of fetal brain development. During fetal maturation, this space undergoes dynamic changes in both shape and size and evolves in response to the growth and maturation of the fetal brain.

The fetal SAS has yet to be studied extensively, though some existing studies can be found on this matter. Recent research, including work conducted by our colleagues, has contributed insight to this interesting topic. Specifically, our colleagues determined the normal range of MR imaging-derived SAS size in fetuses according to gestational age (GA) [4]. This research provided baseline data for normal SAS dimensions in healthy fetuses throughout different stages of fetal development. Other studies have described the SAS measurements in the prenatal period, utilizing transabdominal ultrasound and transvaginal ultrasound, and another study used MR imaging in 2005 [5,6]. In addition, there is also research assessing and discussing relationships between the SAS and other aspects of fetal development, such as the fetal head circumference (HC). These relationships underline the importance of the SAS not just as an anatomical structure but also as a dynamic component of the CNS that interacts with other developmental metrics.

We believe that this is an important matter to study, and that the existing literature has only begun to pave the way to understanding the role and implications of the SAS in fetal development. The relationship between the SAS and the HC is key, as the HC serves as one of several key sonographic measurements of the developing fetus, presenting vital information regarding the fetus’s growth and development. HC measurements are used for pregnancy dating and for the estimation of GA once the crown rump length (CRL) exceeds 84 mm, and is also an indicator of fetal pathologies [7,8,9].

Various fetal pathologies have even been defined by HC measurement. For example, microcephaly is defined as a fetal HC that is >2 SD below the mean for GA, though the definition is not standardized [10]. The incidence of microcephaly varies according to the specific definition used, the population, and the methodology of diagnosis, ranging between 1.3 and 150 per 100,000 births [11]. Microcephaly can either be a single clinical sign or one manifestation of a whole syndrome (several examples are trisomy 21, 18 or 13). Moreover, microcephaly has various etiologies, including congenital infections (Zika virus, toxoplasmosis, rubella virus, cytomegalovirus, herpes virus, syphilis, HIV, etc.), environmental factors (such as exposure to radiation and alcohol) and acute ischemic injury [12].

Macrocephaly, a common pathology affecting 2% of pregnancies, is defined as a fetal HC that is >2 SD above the mean for GA [13]. Fetal macrocephaly has various etiologies: some are genetic and some are even syndromic, while in most cases, the etiology remains unknown [14]. Consequently, prenatal macrocephaly has various prognoses, affected not only by the etiology but also by the degree of head enlargement, parental head circumferences and associated CNS and extra CNS findings [13].

Another interesting fetal pathology is fetal growth restriction (FGR). There is a broad consensus regarding the definition of FGR according to several different national guidelines, differing by whether FGR is defined by EFW alone or if AC measurements are included as well, wherein the cutoff is used to define the abnormal growth (AC cutoff of less than the 10th percentile or less than the 5th percentile), and whether abnormal Doppler findings are incorporated into the definition or not [15,16,17,18]. According to SMFM guidelines in October 2020, the recommended definition of FGR includes either ultrasonographic EFW or AC below the 10th percentile for gestational age [19]. Furthermore, FGR can be classified as either moderate or severe, depending on the percentile, and as symmetrical FGR (featuring a small HC) or the more common asymmetrical FGR (featuring a relatively preserved HC) [20].

Although fetal ultrasound is the screening tool of choice and is the current standard of care in the United States, and although it is believed that most congenital brain abnormalities may be detected first via ultrasound [21,22,23], in our study, we chose to measure the SAS width using MRI for several important reasons. First, studies have presented overdiagnosis involved with diagnosing pathologies such as microcephaly at birth using fetal sonographic reference ranges. Alternatively, studies have revealed that fetal MRI improves diagnostic accuracy for fetal brain anomalies [24,25,26,27,28]. Additionally, MRI is safe during pregnancy to both the mother and fetus, and exposure to 1.5T non-contrast MR imaging during pregnancy has no harmful effects on long-term neurodevelopmental outcomes up to 6 years after delivery [29,30]. Taking all of this into consideration, we are aware of the disadvantages of fetal MRI, including maternal claustrophobia (with a prevalence of failure due to either claustrophobia or malaise of about 2.1% [31]) and fetal movement artifacts and the search for methods of decreasing these faults. In cases where maternal claustrophobia is an issue or when reduction in fetal movement is critical, benzodiazepines are an option, although a study of 131 fetal MRI scans performed in 2012–2013, where 19 of the cases were performed following vallium (diazepam) administration, concluded that low-dose diazepam did not decrease the fetal motion on MRI [32].

Our study aimed to examine the association between the width of the SAS and various fetal pathologies, as measured via fetal MRI. We were specifically curious to compare between groups of fetal pathologies involving pathological HC values: macrocephaly, microcephaly, and FGR.

## 2. Materials and Methods

### 2.1. Study Design and Study Group

This was a retrospective study of 78 fetuses that underwent T2-weighted fetal brain MRI scans during 29–37 weeks of gestation, between 2011 and 2018. All MRI scans were conducted at Sheba Medical Center, a national tertiary referral center in Israel.

Data regarding medical and obstetrical history, perinatal history, sonographic and MR imaging, and medical follow-up were collected using medical charts. A database was formed using the collected information.

Inclusion criteria were sonographic indications of microcephaly or macrocephaly, singleton pregnancies, MRI scans of sufficient quality enabling precise measurements of the SAS, and delivery at Sheba medical center. The FGR group subjects were selected using ultrasound examinations showing abnormal placental findings, specifically placental insufficiency, and were all 10% according to Dolberg growth curves. Their MRIs were performed as part of a pilot study at Sheba Medical Center, aiming to assess the contribution of fetal brain MRI in the diagnosis and management of IUGR [33]. Exclusion criteria were the termination of pregnancy and an associated major brain anomaly (mild findings with minor prognostic significance were included). All other non-placental etiologies of FGR were excluded.

Microcephaly was defined at all modalities as <3% and/or -2SD. Macrocephaly was defined as >97% and/or +2SD. The data were retrieved in different gestational ages, and medians were calculated. To guarantee that our data were not influenced by GA (week), we chose to refer to the HC z-score as presented by Chervenak et al. [34] rather than the absolute HC value in millimeters. We also converted the SAS measurement from millimeters to centiles for the same reason.

### 2.2. MR Images

Fetal brain MR images were obtained using a 1.5T system (Optima MR450w with GEM Suite; GE Healthcare). Examination protocol consisted of single-shot fast spin-echo T2-weighted images in three orthogonal planes. T1-weighted fast-spoiled gradient-echo sequences in the axial plane using a half-Fourier technique (number of excitations = 0.53) were performed with the following parameters: section thickness of 3 mm, no gap, and flexible coil (8-channel cardiac coil). The FOV was determined by the size of the fetal head with a range of 240 × 240 mm to 300 × 300 mm; acquisition time was between 40 and 45 s with matrix = 320/224, TE = 90 ms, TR = 1298 ms, and pixel bandwidth = 122 Hz/pixel; additionally, specific absorption rate values = 1.1–1.7 W/kg. DWI sequence and the calculated ADC map were included [4,35,36]. Fetal MRI was performed without curarization and none of the fetuses received any premedication, while 18 of the mothers received 5 mg of Diazepam as premedication. According to our data, of those 18 who received benzodiazepines, 61% had no movement artifacts, while 28% displayed movement artifacts. In total, 18 cases had movement artifacts recorded on the MRI report: 7 (of 27) in the macrocephaly group, 3 (of 27) in the microcephaly group, and 8 (of 24) in the FGR group. It is important to note that said artifacts did not affect our subarachnoid space measurements.

### 2.3. Subarachnoid Space Width Measurements

**Figure 1:**
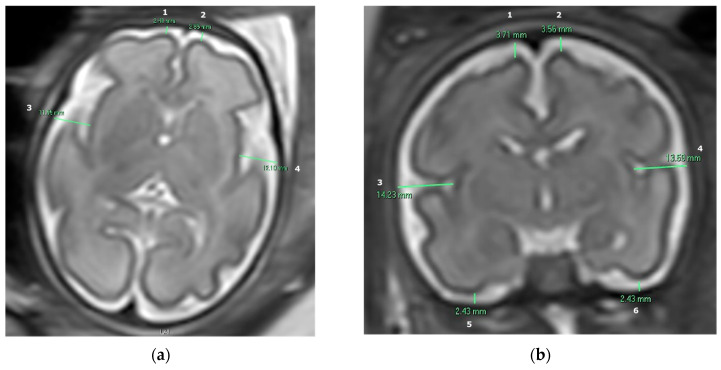
T2-weighted fetal brain MR image demonstrating the measurements of the subarachnoid space in millimeters: (**a**) Axial plane: (1) Right frontal gyrus. (2) Left frontal gyrus. (3) Right insula gyrus. (4) Left insula gyrus. (**b**) Coronal plane: (1) Right frontal gyrus. (2) Left frontal gyrus. (3) Right insula gyrus. (4) Left insula gyrus. (5) Right inferior temporal gyrus. (6) Left inferior temporal gyrus.

The subarachnoid space width was measured in MR images in both axial and coronal planes in ten specific anatomical locations. Measurements were conducted manually in 2D slices using the Carestream Vue PACS system, width measured in millimeters. We obtained four measurements in the axial plane: the right and left frontal gyri and the right and left insula gyri. We also obtained six measurements in the coronal plane: the right and left frontal gyri, the right and left insula gyri, and the right and left inferior temporal gyri, as presented by Figure 1.

To guarantee that our data remain independent of and unaffected by gestational age, we converted the SAS width from millimeters to centiles by comparing our measurements to those of apparently healthy fetuses, previously published by Wandel et al. [4].

### 2.4. Other Measurements

The rest of the parameters discussed in this study (birth weight, STV (supratentorial brain volume), etc.) were collected from medical charts. The STV was measured via 2D MRI measurements and 3D quantitative volumetric MRI measurements using a Matlab-based (MathWorks, Natick, MA, USA) semi-automated software.

### 2.5. Statistical Analysis

The statistical analysis was conducted using SPSS software version 28. Categorical observations were reported as percentages. Continuous variables were reported as median percentiles and IQR. Variables that were not normally distributed were evaluated and compared using the Kruskal–Wallis test. Values that were statistically significant in the Kruskal–Wallis test were then evaluated and compared using the Mann–Whitney U test. All statistical tests were two-tailed. A minimum of 66 fetuses were necessary to provide a power of 80% to detect significant differences, with an effect size of 0.4 (f = 0.4) at a significance level of 5%. 

## 3. Results

In our study, 78 fetuses who met the inclusion criteria were included in our final measurements and categorized into three study groups, as presented in Table 1.

A total of 43 (55%) of the study subjects were male and 35 (45%) were female. The mean GA ± standard deviation (SD) of fetal MRI performance was 34.2 ± 2.2 weeks. The mean birth weight ± SD was 2765.4 ± 910.2 g. The median HC (IQR) in millimeters (measured via ultrasound on the same week as the fetal MRI): 293 (282–302), 334 (323–343), 279 (266–293) for microcephaly, macrocephaly, and FGR accordingly (*p*-value < 0.001). The SAS was notably larger in the macrocephaly group, and markedly smaller in the FGR group.

As previously explained, measurements of the subarachnoid space were collected in ten specific anatomical locations—four in the axial plane and six in the coronal plane (presented in Figure 1)—and two analyses were conducted: one between the macrocephaly and microcephaly groups, and another between the microcephaly and FGR groups.

### 3.1. Macrocephaly Versus Microcephaly

**Figure 2:**
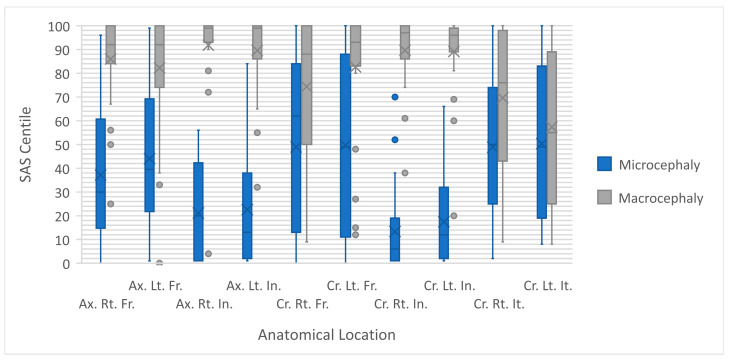
Comparison between the subarachnoid space centiles in the microcephaly (blue) and macrocephaly (gray) groups, as measured in ten anatomical locations: Ax indicates axial; Cr, coronal; Rt, right; Lt, left; Fr, frontal gyrus; In, insular cortex; It, inferior temporal gyrus.

Data regarding the SAS centiles of these two study groups, including the statistical comparison between these groups, are presented in Table 2 and Figure 2.

The median SAS centiles were markedly smaller in the microcephaly group of fetuses (blue in Figure 2) across all ten anatomical locations (all statistically significant differences, except for coronal left inferior temporal gyrus). A significant number of outliers are seen for the macrocephaly group (gray in Figure 2), yet comparison is applicable.

The greatest differences between SAS centiles were found in the insula gyri in both the axial and coronal planes, specifically the coronal right insula gyrus (difference was significant with *p*-value < 0.001). The difference between the groups was also impressive in the axial left insula gyri (difference was significant with *p*-value < 0.001). The anatomical location where the SAS centile values of the two groups exhibited the greatest similarity was the coronal left inferior temporal gyrus, but as previously mentioned, the difference was not statistically significant (*p*-value = 0.457).

Of note, all but two subjects in these two groups had their HC measured at birth using a tape measure, converted later to centiles. A total of 63% of the macrocephaly group subjects had an HC in the macrocephalic range at birth (≥97th percentile, corresponding the threshold of +2 SDs) and 56% of the microcephaly group subjects had an HC in the microcephalic range at birth (≤3rd percentile, corresponding to the threshold of −2 SDs).

### 3.2. Microcephaly Versus FGR

**Figure 3:**
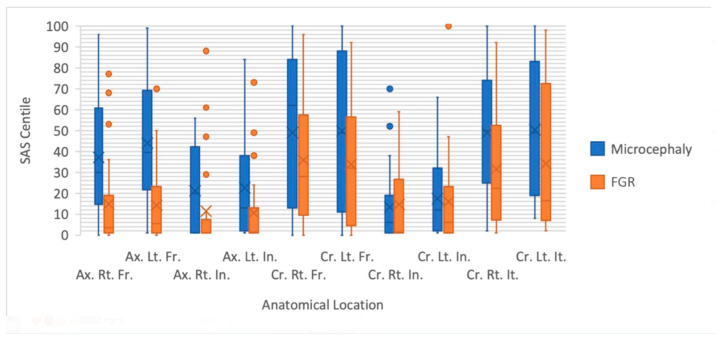
Comparison between the subarachnoid space centiles in the microcephaly (blue) and FGR (orange) groups, as measured in ten anatomical locations: Ax indicates axial; Cr, coronal; Rt, right; Lt, left; Fr, frontal gyrus; In, insular cortex; It, inferior temporal gyrus.

As presented in Table 3 and Figure 3, the median SAS centiles of the microcephaly group of fetuses (presented in blue) are larger than those of the FGR group (presented in orange) across all ten anatomical locations (all statistically significant except for coronal frontal gyri and coronal insula gyri).

Of the anatomical locations measured, the maximal difference between the groups was found in the frontal gyri of both planes: axial left frontal gyri (statistically significant difference with *p*-value < 0.001) and coronal right frontal gyri (no statistical significance, *p*-value = 0.157).

The anatomical location with the greatest similarity in median centiles of these two groups was the coronal right insula gyrus (no statistical significance).

It was interesting to see that in the microcephaly group, unlike in the FGR group, the STV centiles were smaller whilst the SAS centiles were larger. This is true although the HC was not statistically different between the two groups.

## 4. Discussion

The SAS is a marker of fetal brain development, and the most exact noninvasive technique to date that is used to assess the structures of the developing fetal brain is MRI [37,38]. MR imaging can confirm US-positive findings and even provide additional information that can change clinical management in fetuses [39].

Numerous studies have focused on comparing prenatal US with prenatal MRI, examining the strengths and limitations of each to gain insight to their relative diagnostic effectiveness. Only a fraction of them have pinpointed brain imaging. A study that aimed to assess an agreement among prenatal US and MRI (specifically sonographic head circumference (HC) and MRI measurements of fetal supratentorial volume) and to compare these measurements to the HC at birth found that sonographic HC, OFD on 2D MRI, and STV on 3D MRI were all found to be correlated with the HC at birth (Rs = 0.865, *p* < 0.001; Rs = 0.816, *p* < 0.001; Rs = 0.825, *p* < 0.001, respectively) [40]. The findings of the study suggest that although no major difference was found among the modalities, the main advantage of MRI in the evaluation of the fetal brain might be in the diagnosis of subtle structural malformations.

Although ultrasound is common practice during routine pregnancies, this imaging method, transabdominal ultrasound in particular, has shown some technical limitations in evaluating the fetal brain. For instance, transabdominal ultrasound has been associated with technical difficulties in obtaining images of the brain in the coronal plane. A study by Corbacioglu et al. showed that visualizing brain dimensions in the coronal section (specifically sinocortical width and craniocortical width) via transabdominal ultrasound is not easy and that measurements of these areas might be inaccurate as they cannot be seen clearly [41]. Furthermore, multiple studies have illustrated specific challenges in accurately evaluating the SAS using ultrasound, such as the calcification of the fetal calvaria at specific weeks of gestation [42,43].

In contrast, the ability of MRI to detect and identify brain abnormalities that were not previously detected by ultrasound and to offer valuable data that confirm or complement the sonographic findings and potentially alter patient management [44] has led to a greater use of fetal MRI, yet it is not a routine part of all pregnancies as of today. Fetal MRI is typically performed only when certain indications arise, specifically when abnormalities that warrant further investigation are detected via ultrasound. For this reason, our retrospective work lacked a group of normocephalic fetuses, which would have otherwise provided a valuable control group.

When fetal MRI is indicated, the SAS may be assessed and precisely measured. In such cases, it is essential to assess the SAS width in several anatomical locations and multiple imaging planes to accurately distinguish between various benign changes in the SAS size and pathologic brain volumes [45].

### 4.1. Macrocephaly Versus Microcephaly

Macrocephaly has been linked to several pediatric pathologies and may be associated with obstetrical emergencies due to cephalopelvic disproportion [46]. Hence, the evaluation of head circumference is important as an indicator for a cesarean delivery [47].

Macrocephaly may be associated with different SAS widths, making it interesting and even important to measure the SAS in these cases. One of the most common causes of macrocephaly is idiopathic external hydrocephalus: a rapid increase in HC combined with enlarged SAS as seen on neuroimaging, with normal or only moderately enlarged ventricles [48]. In cases where the measured SAS is larger than normal SAS values, a distinction should be made between benign SAS enlargement, such as in external hydrocephalus (a condition that usually resolves without intervention by 2–3 years of age), and pathologic brain atrophy (a pathology with detrimental clinical significance) [45]. Thus, measuring the SAS may help to determine correct management in macrocephaly cases.

Regarding microcephaly, there is clear evidence of the clinical importance of diagnosing this fetal pathology. Extensive studies on patients in mental institutions have shown a close correlation among microcephaly, micrencephaly, and intellectual disability when the head is more than 3D below the normal values [49]. The ability to analyze the fetal HC is critical in clinical practice, particularly for making informed decisions regarding pregnancy termination. However, it is important to note that sonographic criteria for microcephaly vary widely. None of the criteria are ideal as they all have poor performance for predicting adverse neonatal outcome [49]. It is detrimental to assess whether the small HC involves a small brain size or not, and evaluating the SAS can be helpful in these situations.

All in all, optimizing our ability to accurately diagnose fetal microcephaly and macrocephaly is essential since overdiagnosis may cause the unjustified termination of pregnancy, while the misdiagnosis of fetal HC could result in problematic and even dangerous labor. Measurements of the SAS can be a great asset in this particular field of practice.

According to our results, the SAS is larger and wider in the macrocephaly group than in the microcephaly group. Upon reviewing the differences between the measured anatomical locations, it becomes apparent that the insula gyrus—on both left and right sides and in both coronal and axial planes—shows the most significant variation in SAS centiles among the study groups. One clinical correlation found in the literature between a small insular cortex and fetal microcephaly is polymicrogyria, a cortical malformation that can be restricted to the insula, and is commonly seen in microcephalic children [50].

Our study limitation was the fact that only 27 of the 78 fetuses (35%) were within the pathological HC range (Chervenak Z Score SD ≥ +2 or ≤−2). The microcephaly group includes fetuses that had ultrasonic indications of microcephaly. In our work, the median HC Chervenak Z Score SD did not meet the definition of microcephaly, and only 11/27 fetuses (41%) in this group had an HC Chervenak Z Score smaller than 2 SD. In the macrocephaly group, only 5/27 fetuses (19%) had an HC Chervenak Z Score greater than 2 SD. Potential future studies could add to the inclusion criteria of pathological-range HC to draw a more concise conclusion regarding the association between the SAS width and HC-based fetal pathologies.

### 4.2. Microcephaly Versus FGR

Our study demonstrates the difference in SAS width between these two pathological states that both present with a small HC compared to normal values, though their etiologies differ.

According to our results, while the FGR group presents a rather preserved brain volume, microcephalic fetuses exhibit a definite pathological brain volume. This was expected and can be explained by the literature. Microcephaly often coincides with micrencephaly, a small brain volume. On the other hand, while fetuses with FGR (symmetrical FGR) have small HCs and a low estimated fetal weight, their brain volume features a “brain-preserving effect” accounting for their near normal brain size [51]. Taking these facts into consideration, we expected the SAS to be larger in the microcephaly group due to their smaller brain size. As expected, a comparison between these two groups of fetuses demonstrated larger SAS centiles (in all ten anatomical locations) along with smaller STV centiles (two values had no statistical difference). It seems that, according to our results, the difference in SAS size can account (at least in part) for the many clinical differences between these two pathological states.

Furthermore, previous work on fetuses with FGR presented fetuses with FGR with specifically reduced frontal lobe growth when compared with appropriately grown fetuses [52], which can account for the considerable difference in SAS in the frontal gyri in our study.

In connection with the previous topics, there are additional fetal pathologies that exhibit both small HC and reduced feal growth. One example is fetuses with congenital heart defects who have been found to present with FGR and impaired head growth, associated with poor neurodevelopmental outcomes postnatally [53,54,55]. It would be interesting to study an association between the subarachnoid space and congenital heart disease in fetuses, and to assess the ratio between the subarachnoid space and the fetal brain volume in this specific subgroup.

## 5. Conclusions

In conclusion, our study reveals a statistically significant association between the SAS width and fetal pathologies, paving the way to understanding the roles and implications of the SAS and fetal development. As an indicator of brain development, associating the SAS width measurement with fetal pathologies can provide both parents and clinicians vital information regarding sdeveloping fetuses. It would be interesting to correlate the results of our study with information regarding the postnatal neurological status of the fetuses, information which we did not include in the current methodology of our study. We suggest examining an association between the SAS and other fetal pathologies in future work.

## Figures and Tables

**Table 1:** Demographic and clinical characteristics, and measured and calculated values of the study subjects.

	Macrocephaly	Microcephaly	FGR	*p*-Value
Cases (n)	27	27	24	
Gender–Male (%)	88.9	14.8	62.5	<0.001
GA at MRI scan (weeks)	34.14 (32.57–35.14)	35.86 (34.00–36.86)	33.07 (31.5–36.0)	0.002
GA at birth (weeks)	38.43 (37.86–39.57)	38.93 (38.14–39.64)	36 (34–37)	<0.001
Birth weight (grams)	3596 (3320–4005)	2817.50 (2636.25–3055.25)	1619 (1398–1930)	<0.001
Birth weight (Dolberg centile)	81 (71–96)	23 (18.5–44.0)	3 (1–6)	<0.001
HC Chervenak Z Score SD	1.44 (1.31–1.84)	−1.90 ((−2.21)–(−1.37))	−1.64 ((−2.45)–(−1.26))	<0.001
STV (mm^3^)	251,710 (229,659–280,723)	192,251 (149,988–213,750)	185,268.51 (139,408.72–212,800.86)	<0.001
STV centiles	96 (70–99)	2 (0.5–9)	5 (3.0–19.4)	<0.001
SAS index (mm)	91.55 (86.35–101.05)	59.46 (50.00–66.91)	53.21 (49.71–59.10)	<0.001

Data are presented as median (interquartile range). Note: HC indicates head circumference; GA, gestational age; SAS, subarachnoid space; STV, supratentorial brain volume; SAS, subarachnoid space.

**Table 2:** Comparison between the subarachnoid space centiles of the macrocephaly and microcephaly groups.

Plane	Location	Macrocephaly	Microcephaly	*p*-Value
Axial	Rt. Fr.	92 (84–100) **	30 (14.75–60.75) *	<0.001
	Lt. Fr.	92 (74–100) **	39.50 (21.75–69.25)	<0.001
	Rt. In.	99 (93–100) **	20 (1.00–42.25) **	<0.001
	Lt. In.	99 (86–100) **	13 (2–38) **	<0.001
Coronal	Rt. Fr.	88 (50–100) **	62 (13–84)	0.009
	Lt. Fr.	93 (83–100) **	49 (11–88)	<0.001
	Rt. In.	97 (86–100) **	6 (1–19) **	<0.001
	Lt. In.	96 (89–99) **	12 (2–32) **	<0.001
	Rt. It.	76 (43–98) **	50 (25–74)	0.011
	Lt. It.	55 (25–89)	49 (19–83)	0.457

Data are presented as median (interquartile range). Note: Rt indicates right; Lt, left; Fr, frontal gyrus; In, insular cortex; It, inferior temporal gyrus. *—Correlation is significant at the 0.05 level (2-tailed). **—Correlation is significant at the 0.01 level (2-tailed).

**Table 3:** Comparison between the subarachnoid space centiles of the microcephaly and FGR groups.

Plane	Location	Microcephaly	FGR	*p*-Value
Axial	Rt. Fr.	30 (14.75–60.75) *	3.5 (1–19) **	0.003
	Lt. Fr.	39.50 (21.75–69.25)	5.5 (1.00–23.25) **	<0.001
	Rt. In.	20 (1.00–42.25) **	1 (1.00–7.50) **	0.038
	Lt. In.	13 (2–38) **	1.5 (1–13) **	0.017
Coronal	Rt. Fr.	62 (13–84)	28 (9.5–57.5) *	0.157
	Lt. Fr.	49 (11–88)	32 (4.5–56.5) *	0.117
	Rt. In.	6 (1–19) **	1.5 (1.00–26.75) **	0.493
	Lt. In.	12 (2–32) **	6 (1.00–23.25) **	0.457
	Rt. It.	50 (25–74)	22.5 (7.25–52.5) **	0.035
	Lt. It.	49 (19–83)	16.5 (7.00–72.50) *	0.035

Data are presented as median (interquartile range). Note: Rt indicates right; Lt, left; Fr, frontal gyrus; In, insular cortex; It, inferior temporal gyrus. *—Correlation is significant at the 0.05 level (2-tailed). **—Correlation is significant at the 0.01 level (2-tailed).

## Data Availability

The raw data supporting the conclusions of this article will be made available by the authors on request.
